# Overexpressed immunoglobulin-like transcript (ILT) 4 in lung adenocarcinoma is correlated with immunosuppressive T cell subset infiltration and poor patient outcomes

**DOI:** 10.1186/s40364-020-00191-7

**Published:** 2020-04-29

**Authors:** Qing Li, Juan Li, Shuyun Wang, Jingnan Wang, Xiaozheng Chen, Dongmei Zhou, Yuying Fang, Aiqin Gao, Yuping Sun

**Affiliations:** 1 Department of Oncology, Jinan Central Hospital affiliated to Shandong University, Jinan, 250013 Shandong P. R. China; 2 Department of Oncology, Yantaishan Hospital, Yantai, 264000 Shandong P.R. China; 3 Department of Oncology, Jinan Central Hospital affiliated to Shandong First Medical University, Jinan, 250013 Shandong P. R. China

**Keywords:** Immunoglobulin-like transcript 4, Lung adenocarcinoma, T cell subset, Immunosuppression

## Abstract

**Background:**

The poor response to current PD-1/PD-L1 inhibitors in lung cancer patients requires development of novel immunotargets. Immunoglobulin-like transcript (ILT)4 is an immunosuppressive molecule mainly expressed in myeloid innate cells. Recent studies showed that ILT4 was highly expressed in multiple malignant cells and regulated tumor biologies including proliferation, invasion and metastasis. However, the immunomodulatory role of tumor cell-derived ILT4 is unclear. Here we aimed to analyze the correlation of tumor cell ILT4 expression with T cell infiltration and subset distribution, illustrate ILT4-regulated immunosuppressive microenvironment, and raise tumor cell-derived ILT4 as a novel immunotherapeutic target and prognostic biomarker for lung adenocarcinoma (LUAD) patients.

**Methods:**

We collected the tissue samples and corresponding clinicopathological data from 216 primary LUAD patients. Using immunohistochemical staining and public database analyses we investigated the relationship between ILT4 expression and different T cell subset density as well as patient outcomes.

**Results:**

Enriched ILT4 expression in tumor cells of LUAD tissues indicated reduced T cell infiltration in the tumor microenvironment (TME), advanced diseases and poor patient overall survival (OS). Further T cell subset analyses revealed that ILT4 expression was correlated with decreased CD8^+^T cell and increased Treg frequency in both cancer nest and stroma, but not with altered CD4^+^T cell frequency. High ILT4 level combined with low CD8^+^T cell/high Treg density predicted markedly poorer clinical outcomes compared with any of these biomarkers alone.

**Conclusions:**

Tumor cell-derived ILT4 is correlated with immunosuppressive T cell subset infiltration and poor clinical outcomes, and might be a potential immunotherapeutic target and prognostic biomarker for LUAD patients. Combined ILT4 expression and CD8^+^ T cell/Treg frequency in tumor infiltrating lymphocytes (TILs) are stronger predictors for patient outcomes.

## Background

Lung cancer is the leading cause of cancer morbidity and mortality worldwide [1]. As the most frequent histological subtype, the incidence of LUAD still trends to increase in most countries [2]. The multidisciplinary comprehensive treatment including chemotherapy, radiotherapy and driver gene-targeted therapy has reached the bottleneck with a 5-year survival rate of 21% [3]. Immune checkpoint blockade (ICB) in recent years has revolutionized the anti-tumor therapy and is considered as a potential curative strategy for malignancies [4]. However, the objective response rate of single PD-1/PD-L1 inhibitors in lung cancer is merely 20% [4]. Except for the inadequate patient selection and tumor intrinsic hypoimmunogenicity, the complex immunosuppressive microenvironment, which contains inhibitory immunocytes, cytokines and metabolites as well as decreased TIL number and functionality, presents a major hurdle to T cell immunity and effective ICB therapy [5, 6]. Therefore, the development of novel immunotargets and treatment are urgently needed to break the suppressive barrier in anti-tumor immunotherapy.

Immunoglobulin-like transcript (ILT) 4, also named lymphocyte immunoglobulin-like receptor B (LILRB) 2, LIR-2, monocyte/macrophage immunoglobulin-like receptor 10 (MIR-10), or CD85d, is an immunosuppressive receptor mainly expressed in myeloid innate cells including dendritic cells (DCs), monocytes, macrophages and neutrophils [7–9]. ILT4 expression in these cells represents their suppressive phenotypes and inhibits their immune response [10]. Thus, ILT4 plays important roles in the immune pathologies such as fetal-maternal tolerance, allograft rejection and infectious and autoimmunity diseases [10]. In 2008, we firstly reported that ILT4 was enriched in tumor cells of non-small cell lung cancer (NSCLC) and predicted advanced tumor stages [11]. Subsequent studies by us and other groups showed that tumor cell-derived ILT4 directly regulated their proliferation, invasion, migration and epithelial-mesenchymal transition (EMT) and promoted tumor progression [12–14]. Recently, other groups identified the expression of ILT4 and its mouse homologue paired Ig-like receptor (PIR-B) in immunocytes of the tumor microenvironment (TME) including myeloid-derived suppressor cells (MDSCs), tumor-associated macrophages (TAMs) and hemopoietic stem cells (HSCs) [15, 16]. ILT4 in these cells supported M2 polarization of MDSCs and TAMs, and created immunosuppressive microenvironment [15, 16]. So for the first time, we proposed the concept that “ILT4 is a potential checkpoint molecule in tumor immunotherapy” [10]. However, how tumor cell-derived ILT4 controls T cell subset infiltration and their spatial distribution is still unclear.

In the current study, we found that enriched ILT4 expression in tumor cells was correlated with decreased T cell infiltration in the TME and progressive diseases of LUAD patients. Further subset analyses revealed that higher ILT4 expression was connected to decreased CD8^+^T cell and increased FOXP3^+^ regulatory T cell (Treg) infiltration in both cancer nest and stroma. Tumor cell-derived ILT4 together with decreased CD8^+^T cells or increased Tregs were stronger negative prognostic indicators for LUAD patients compared with ILT4 expression or CD8^+^T cell/ Treg infiltration alone. Our work gave a cue that ILT4 might regulate suppressive T cell subset infiltration and tumor immune escape. Meanwhile, we provided more predictive prognostic biomarkers for LUAD patients.

## Materials and methods

### Patients and tissue samples

On the approval of the review board and ethics committee, 216 lung adenocarcinoma specimens were collected from newly diagnosed patients in Yantaishan hospital (Yantai, China) from 2008.01 to 2016.01. All the patients underwent primary surgery or biopsy without preoperative treatment including chemotherapy, radiotherapy or immunotherapy. Among the 216 cases, 113(52.31%) were male and 103(47.69%) were female. The average age was 60.66 (20–85) years old. 124 (57.41%) patients had the tumor diameter of ≥3 cm and 92(42.59%) < 3 cm. According to the TNM classification of International Union against Cancer (UICC) in 2017, 82 (37.96%) patients were determined as stage I, 39(18.06%) as stage II, 52(24.07%) as stage III, and 43(19.91%) patients as stage IV.

### Immunohistochemistry analysis

Paraffin-embedded LUAD tissues were sequentially sectioned into 5 pieces with the thickness of 4 μm. The slides were first deparaffinized in xylene and rehydrated in gradient ethanol. Antigen retrieval was performed by microwave oven at 95 °C for 10 min in Tris-ethylenediaminetetraacetic acid buffer with the pH 6.0. Then the sections were soaked in 3% hydrogen peroxide solution for 10 min to eliminate endogenous peroxidase and subsequently in goat serum to block the non-specific antigens. Afterwards, the slides were incubated in corresponding primary antibodies overnight at 4 °C for complete immune binding. The primary antibodies were as follows: anti-ILT4 antibody (1:25, Origene, Cat#TA349368), anti-CD3 antibody (1100, Proteintech, Cat#17617–1-AP), anti-CD4 antibody (1100, Abcam, Cat#ab133616), anti-CD8 antibody (ready-to-use, Zhongshan Jinqiao Biotechnology Co. Ltd., Cat#ZA-0508), anti-FoxP3 antibody (1,25, Abcam, Cat#ab20034). Sections incubated with mouse or rabbit IgG were applied as negative control. To detect the primary antibody binding, the sections were incubated with Elivision Plus Polymer Horseradish Peroxidase (Rabbit /Mouse) IHC Kit for 25 min and then streptavidin-conjugated peroxidase for 25 min at room temperature. Finally, sections were visualized with 3,3′-diaminobenzidine solution (MXB) and counterstained with hematoxylin.

For each slide, at least five fields were reviewed at × 400 magnification by two independent investigators in a randomized, double-blind manner. The expression of ILT4 in tumor cells was evaluated by both positive cell ratio (proportion score) and staining intensity (intensity score). The proportion scores were estimated as follows: 0 = none; 1 = less than 25%; 2 = 25–75%; 3 = greater than 75%. The intensity scores were estimated as follows: 0 = none; 1 = weak; 2 = intermediate; 3 = strong. The final score for each patient was expressed as the product of the proportion and intensity scores. Cutoff scores for high and low ILT4 expression were ≥ 4 and < 4, respectively. Different T cell subsets were defined by the frequency of CD3^+^/CD4^+^/CD8^+^/FOXP3^+^ T cells in total lymphocytes. The cutoff values for high and low T cell subset frequency were defined by ≥ median and < median, respectively.

### The correlation analysis of ILT4 expression with patient survival and Treg infiltration in public databases

The online tool of KM-plotter (http://kmplot.com/) database was used to analyze ILT4-based survival in LUAD patients. A total of 720 and 461 LUAD patients from Gene Expression Omnibus (GEO) database were analyzed for progression free survival (PFS) and OS respectively. The best cutoff value were auto-selected for each cohort and all other parameters were at default settings.

Gene expression profiles of LUAD patients were downloaded from GEO dataset (GSE50081; https://www.ncbi.nlm.nih.gov/geo/query/acc.cgi?acc=GSE50081). Gene annotation of GSE50081 was based on the microarray platform GPL570 and 127 LUAD samples were engaged in further study. For the Cancer Genome Atlas (TCGA) cohort, the RNASeq data of pan-cancer were downloaded from UCSC xena (https://xenabrowser.net/datapages/). Five hundred and fifteen samples were enrolled in this cohort. The Treg infiltration score were quantified using the ssGSEA function in R package GSVA [17]. Spearman correlation coefficient was used to evaluate the relevance between ILT4 expression and Treg infiltration.

### Survival follow-up and statistical analysis

The statistical analysis was performed using GraphPad Prism 8.0 software (GraphPad Software Inc., USA). The association between ILT4 expression and T cell subset frequencies/ clinicopathological variables were analyzed using the student two-tailed t test/Fisher’s exact test respectively. The overall survival time were obtained by telephone follow-up of all the patients. The last censor were on February 25, 2020. Kaplan-Meier and the log rank test were used to plot the survival curve. *P* < 0.05 was considered as statistically significant difference.

## Results

### ILT4 expression in LUAD tissues predicted advanced disease stages and poor patient survival

**Fig. 1:**
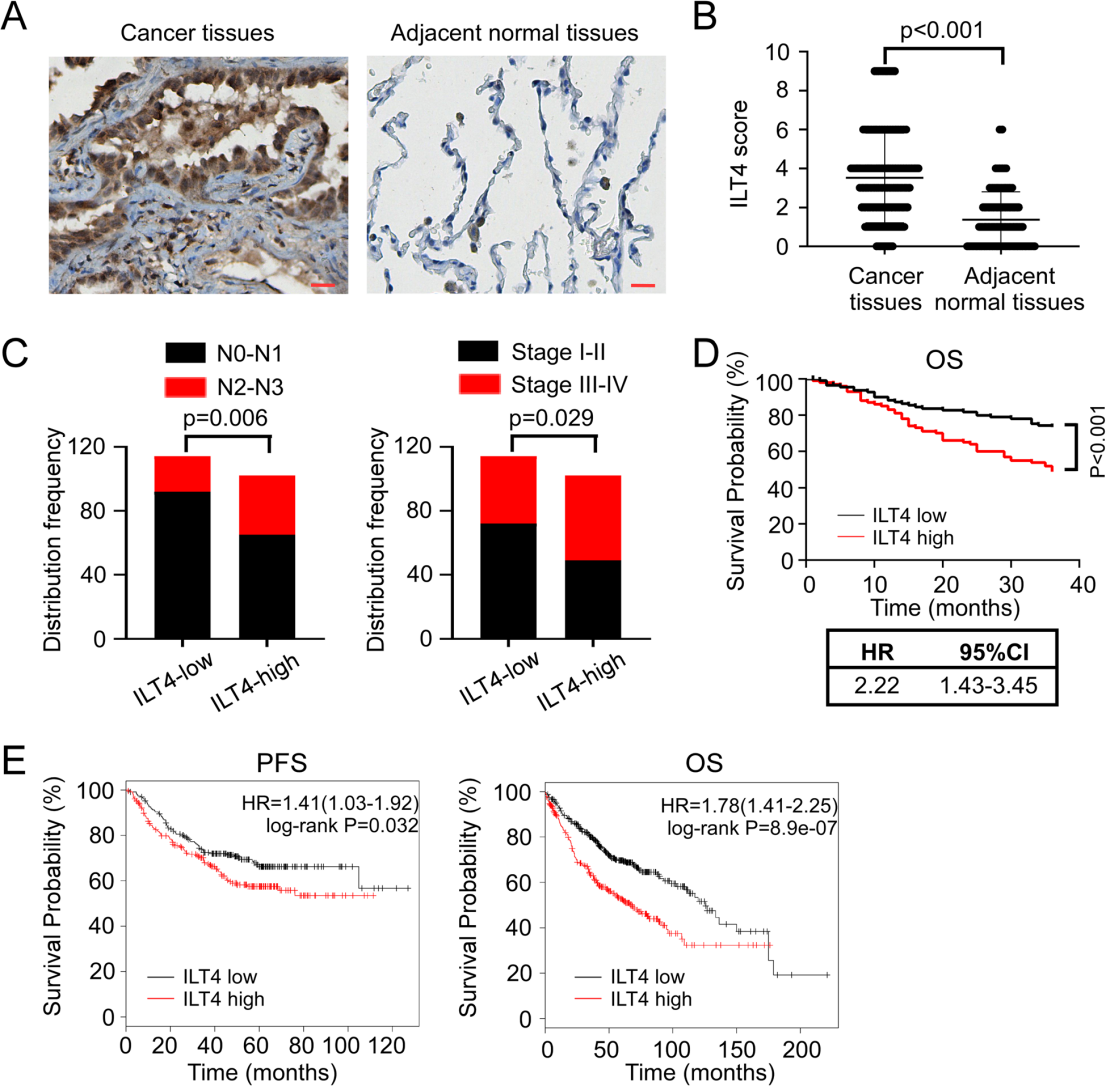
ILT4 expression in LUAD tissues predicted advanced diseases and poor patient survival. **a** & **b** ILT4 expression was significantly higher in tumor cells of LUAD tissues compared with that in adjacent normal tissues by immunohistochemical staining. **a** showed the typical images of ILT4 expression, brown granules were defined as positive staining. **b** showed the statistical results in all patients (*p* < 0.001). Scale bar:20 μm. **c** High ILT4 expression in LUAD tissues was correlated with advanced lymph node metastasis (*p* = 0.006) and TNM stages (*p* = 0.029). The cutoff scores for high and low ILT4 expression were ≥ 4 and < 4 respectively. **d** Patients in ILT4-high group showed markedly poorer OS compared with that in ILT4-low group (*p* < 0.001; HR = 2.22, 95%CI:1.43–3.45). According to the cutoff score, 102 patients were included in ILT4-high group and 114 in ILT4-low group. **e** ILT4 expression levels in human LUAD tissues were negatively associated with patient PFS and OS. Transcriptional expression levels of ILT4 and patient survival information were obtained from the GEO database. 720 and 461 LUAD patients were included for PFS and OS analysis, and the best cutoff values were auto-selected by the online tool of KM-plotter database (*p* = 0.032 for PFS and *p* = 8.9e-07 for OS)

A total of 216 human LUAD samples were examined and the corresponding clinicopathological information were analyzed. According to the brown staining, positive ILT4 expression was mainly identified in the cytoplasm or membrane of the tumor cells, but rarely or minorly in the normal lung epithelial cells adjacent to the tumor lesions (Fig. 1a). The statistical results revealed that ILT4 expression in cancer cells was significantly higher than that in adjacent normal tissues (Fig. 1b). Then we analyzed the correlation between ILT4 expression and patients’ clinicopathological characteristics. We found that compared with ILT4-low group, ILT4-high group displayed more advanced regional lymph node involvement and TNM stages (Fig. 1c). These results are in accordance with our previous findings [12]. We also compared the OS between ILT4-high and -low patients. Kaplan-Meier analysis showed that patients in ILT4-high group had remarkably shortened OS (HR = 2.22, 95%CI:1.43–3.45) (Fig. 1d). Using GEO database, we confirmed that ILT4 expression in LUAD patients predicted poorer patient PFS (HR = 1.41, 95%CI:1.03–1.92) and OS (HR = 1.78, 95%CI:1.41–2.25) (Fig. 1e). Taken together, these results indicated that ILT4 is an adverse prognostic biomarker for LUAD patients and might play critical roles in tumor progression.

### Overexpressed ILT4 in tumor cells was associated with decreased T cell infiltration in LUAD patients

**Fig. 2:**
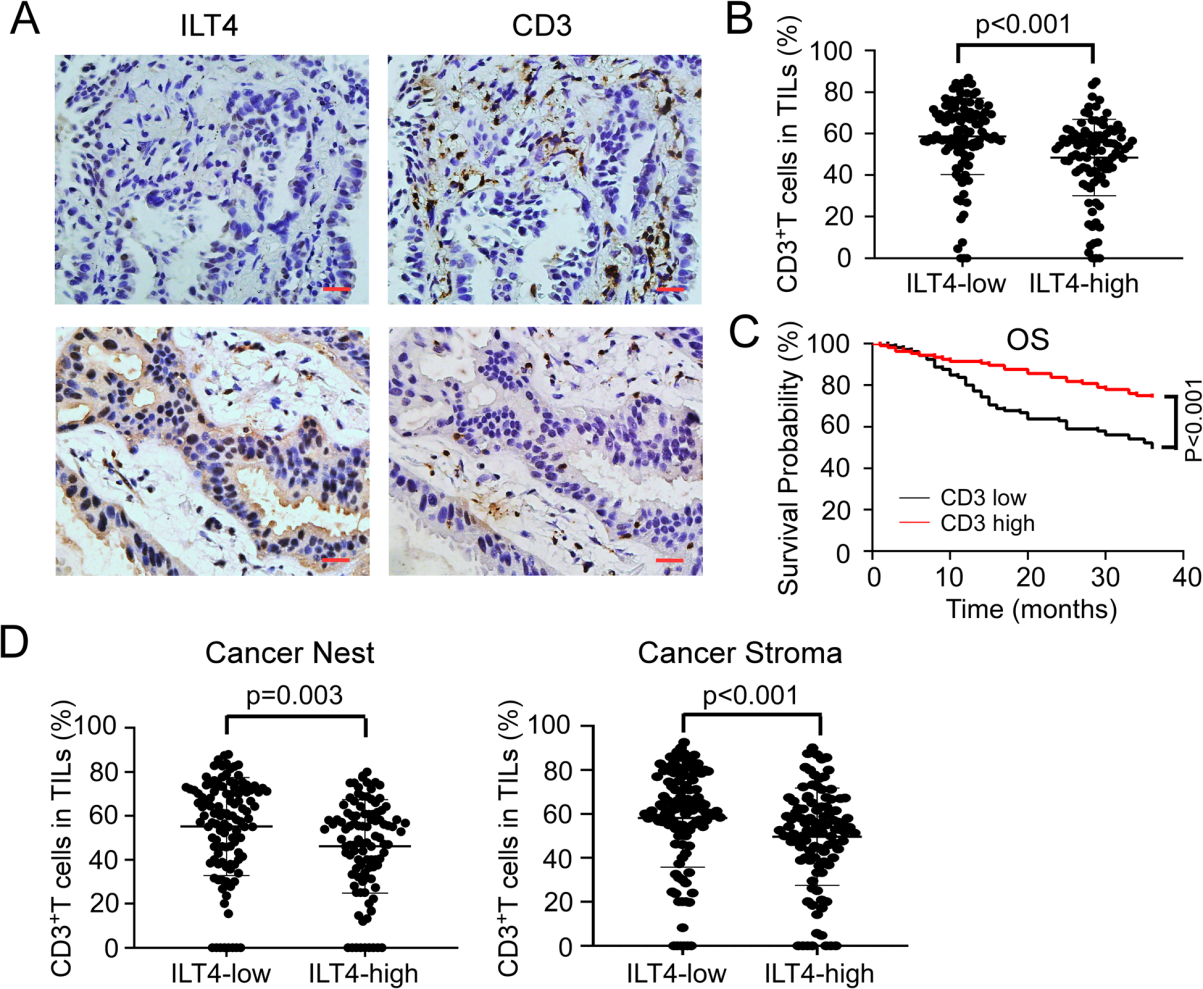
Overexpressed ILT4 in tumor cells was associated with decreased T cell infiltration in LUAD patients. **a** & **b** ILT4-high group displayed fewer CD3^+^T lymphocyte infiltration compared with the ILT4-low counterpart by immunohistochemical staining. **a** showed the typical images of ILT4 expression and infiltrating CD3^+^T cell density. Each paraffin-embedded tissue was sequentially sectioned for ILT4 or CD3 staining respectively. **b** showed the statistical result in all patients (*p* < 0.001). Scale bar:20 μm. **c** Low CD3^+^T cell frequency in TILs indicated poorer patient OS (*p* < 0.001). The cutoff value for high and low CD3 frequency were defined as ≥ median and < median respectively. **d** High ILT4 expression in tumor cells was correlated with reduced CD3^+^T cell proportion both in the cancer nest (*p* = 0.003) and stroma (*p* < 0.001). The cutoff scores for high and low ILT4 expression were the same as in Fig. 1c

ILT4 is a classic immunosuppressive molecule in the myeloid innate cells [10]. However, the immunological function of tumor cell-derived ILT4 is still undetermined. Given that T cell immunity represents the most crucial component in anti-tumor immunity, we evaluated ILT4-regulated T cell infiltration in the TME. We found that the frequency of tumor infiltrating CD3^+^ T cells in ILT4-high tissues was much lower than that in ILT4-low tissues (Fig. 2a, b). To address the clinical significance of T cell infiltration, we determined patient OS depending on CD3^+^ T cell ratio in TILs. As expected, CD3-high group showed beneficial OS compared with CD3-low counterpart (Fig. 2c). These results suggested that ILT4 might mediate tumor immune escape through inhibition of T cell infiltration. It was reported that direct contact between tumor cells and T cells (defined as immunological synapse) is important for T cell-induced cytotoxicity and memory [18], so we tried to explore whether ILT4 controlled the spatial arrangement of tumor infiltrating T cells. We calculated the frequency of CD3^+^ T cells in both cancer nest and stroma respectively and found that compared with ILT4-low group, ILT4-high group showed significantly reduced CD3^+^ TIL number in both cancer nest and stroma (Fig. 2d), suggesting ILT4-regulated T cell immunity is not dependent on direct cell-cell contact. Collectively, tumor cell-derived ILT4 is associated with impaired T cell infiltration in both cancer nest and stroma, which might subsequently destruct anti-tumor immunity and patient outcomes.

### High ILT4 expression in tumor cells was correlated with reduced CD8^+^T cell and elevated Treg infiltration

**Fig. 3:**
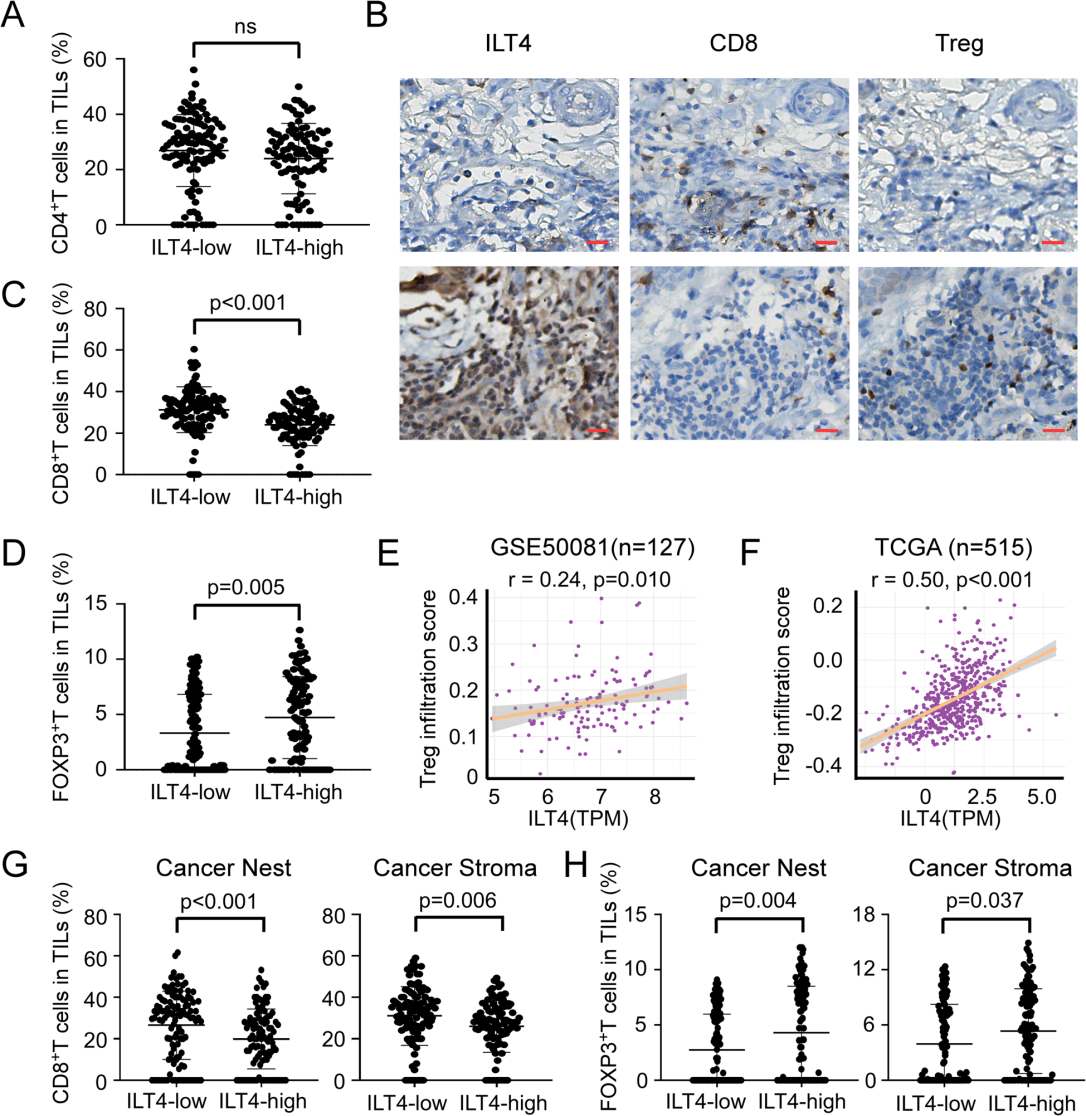
High ILT4 expression in tumor cells was correlated with reduced CD8^+^T cell and elevated Treg infiltration. **a** No significant correlation was found between ILT4 level and CD4^+^T cell proportion in LUAD tissues. The cutoff scores for ILT4-high and -low patients were defined as in Fig. 1c. **b** Patients in ILT4-high group had fewer CD8^+^T and more FOXP3^+^ T lymphocyte infiltration in TME compared with ILT4-low group by immunohistochemical assays. The typical images were presented. Scale bar: 20 μm. **c** & **d** Patients in ILT4-high group showed significantly decreased CD8^+^T (*p* < 0.001) and increased FOXP3^+^ T (*p* = 0.005) cell frequency in TILs. **e** & **f** The transcriptional expression level of ILT4 in LUAD tissues was positively correlated to Treg infiltration by analyses of GEO dataset (GSE 50081, *n* = 127) and TCGA database (*n* = 515). The correlation coefficient(r) was 0.24 and 0.50 respectively. **g** & **h** High ILT4 expression in tumor cells predicted decreased CD8^+^T (**e**) (p < 0.001 for cancer nest and *p* = 0.006 for stroma) and increased FOXP3^+^ T (**f**) (*p* = 0.004 for cancer nest and *p* = 0.037 for stroma) cell frequency in both cancer nest and stroma

Since ILT4 expression is correlated with decreased T cell infiltration, we next want to probe into ILT4-regulated T cell subset composition. We detected tumor infiltrating CD4^+^, CD8^+^ and FOXP3^+^ T cell proportion in TILs of ILT4-high or ILT4-low patients respectively. No difference in CD4^+^ T cell frequency was found between these two groups (Fig. 3a). However, tumor infiltrating CD8^+^ T cells were significantly decreased in ILT4-high group compared with that in ILT4-low group (Fig. 3b, c). In addition, ILT4-high group displayed markedly more abundant FOXP3^+^ Treg cell accumulation (Fig. 3b, d). We also searched the public databases to confirm the relevance of ILT4 expression with immune cell infiltration in LUAD tissues. As expected, both in the GSE50081 dataset from GEO database and in the TCGA database, the transcriptional expression level of ILT4 in tumor tissues was positively correlated with Treg infiltration (Fig. 3e, f). Next we determined the spatial arrangement of these altered T cell subsets. Similar with the result for total T cells, both in cancer nest and stroma, ILT4-high group displayed markedly reduced CD8^+^ TIL (Fig. 3g) and increased Treg density (Fig. 3h). These results suggested that tumor cell-derived ILT4 prevented the infiltration of tumoricidal cytotoxic CD8^+^ T cells and recruited tumor-suppressive Tregs, which are responsible for ILT4-impaired anti-tumor immune response.

### High ILT4 expression in combination with decreased CD8^+^ T cells or increased Tregs are stronger indicators for poor patient outcomes

**Fig. 4:**
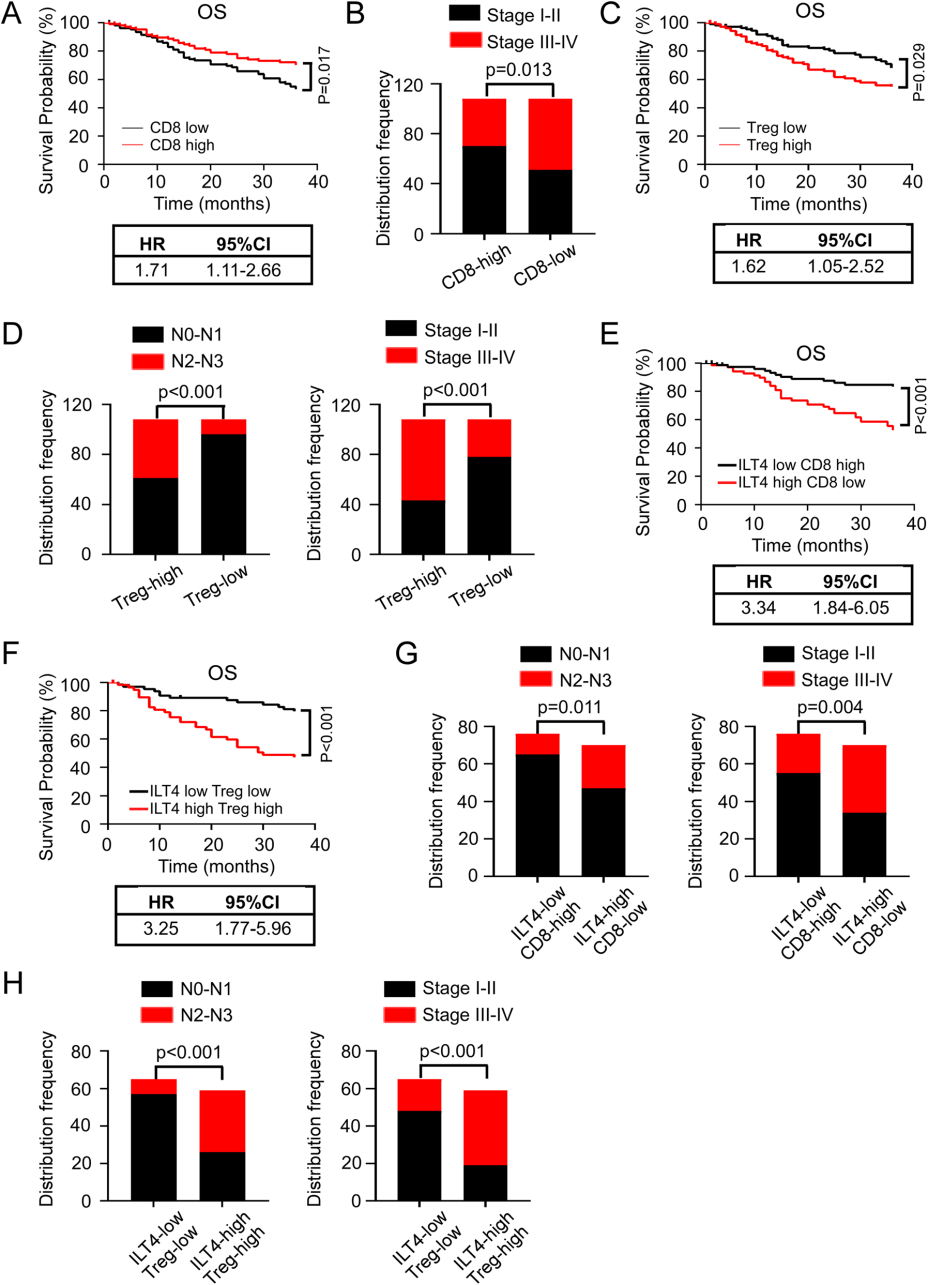
High ILT4 expression in combination with decreased CD8^+^ T cell or increased Treg infiltration are stronger indicators for poor patient outcomes. **a** & **b** Compared with the high counterpart, low CD8^+^T cell proportion in TILs predicted poorer OS (**a**) (*p* = 0.017; HR = 1.71, 95%CI:1.11–2.66) and advanced TNM stages (**b**) (*p* = 0.013) in LUAD patients. The cutoff value for high and low CD8^+^T cell frequency were defined as ≥ median and < median respectively. **c** & **d** Compared with the low counterpart, high Treg proportion in TILs predicted poorer OS (**c**) (*p* = 0.029; HR = 1.62, 95%CI:1.05–2.52), advanced lymph node involvement and TNM stages (**d**) (both *p* < 0.001) in LUAD patients. The cutoff value for high and low Treg frequency were defined as ≥ median and < median respectively. **e** & **f** High ILT4 level combined with low CD8^+^T cell/high Treg frequency were stronger predictors for poor patient OS. Compared with the corresponding counterpart controls, ILT4-high+CD8-low (**e**) (*p* < 0.001; HR = 3.34, 95%CI: 1.84–6.05) and ILT4-high+Treg-high (**f**) (*p* < 0.001; HR = 3.25, 95%CI:1.77–5.96) patients showed statistically shortened OS. It is noteworthy the combined groups had higher HR values compared with any of the single marker groups (HR = 2.22 for ILT4 alone, HR = 1.71 for CD8^+^T cell alone and HR = 1.62 for Treg alone). The cutoff value for ILT4/CD8^+^T cell/Treg high and low were defined as in Fig. 1c, Fig. 4a and c. **g** & **h** Compared with the corresponding counterpart controls, ILT4-high+CD8-low (**g**) (*p* = 0.011 for LN involvement and *p* = 0.004 for TNM stages) and ILT4-high+Treg-high (**h**) (*p* < 0.001 for both LN involvement and TNM stages) patients showed more advanced lymph node involvement and TNM stages

We have discovered that tumor cell-derived ILT4 might orchestrate CD8^+^ T cell and Treg infiltration in the TME. However, the functional value of CD8^+^ T cell/Treg density in LUAD patients remained to be fully elucidated. We analyzed the clinicopathological features and OS in LUAD patients depending on CD8^+^T cell/Treg frequency. The results showed that CD8^+^T cell-low group had poorer OS and more advanced TNM stages (Fig. 4a, b) compared with its counterpart control. In contrast, patients in Treg-low group showed beneficial OS, less lymph node involvement and earlier clinical stages (Fig. 4c, d). These results verified the positive/negative predictive role of CD8^+^ T cell/Treg infiltration in patient outcomes respectively.

Next we wanted to determine the prognostic value of ILT4-regulated CD8^+^T cell/Treg infiltration. The patients were classified into 4 subgroups based on the level of ILT4 expression and infiltrating CD8^+^ T cells: ILT4^high^CD8^high^ (*n* = 32), ILT4^high^CD8^low^ (*n* = 70), ILT4^low^CD8^high^ (*n* = 76) and ILT4^low^CD8^low^ (*n* = 38). Similarly, four subgroups were generated as well based on ILT4 expression and Treg density: ILT4^high^Treg^high^ (*n* = 59), ILT4^high^Treg^low^ (*n* = 43), ILT4^low^Treg^high^ (*n* = 49) and ILT4^low^Treg^low^ (*n* = 65). Using Kaplan-Meier survival analysis, we found that ILT4^high^CD8^low^ and ILT4^high^Treg^high^ groups showed significantly shortened OS than ILT4^low^CD8^high^ and ILT4^low^Treg^low^ groups respectively (Fig. 4e, f). It is noteworthy that the hazard rate (HR) of OS based on the combined biomarkers (HR = 3.34 for combined ILT4 and CD8; HR = 3.25 for combined ILT4 and Tregs) were much higher than that based on ILT4(HR = 2.22), CD8^+^T cells (HR = 1.71) or Tregs (HR = 1.62) alone, suggesting that the combined biomarkers are stronger indicators of patient survival. Furthermore, ILT4^high^ combined with CD8^low^/Treg^high^ are solid predictors for advanced regional lymph node involvement and TNM stages (Fig. 4g, h). In summary, these results clearly suggested that ILT4 combined with CD8^+^ T cell or Treg infiltration are stronger indicators for poor patient outcomes.

## Discussion

In recent years, ICB therapy, which targets PD-1/PD-L1 axis to unleash the killing ability of CD8^+^ T cells, has dramatically transformed the therapeutic paradigm for NSCLC [19]. However, in most patients, ICB exerted limited clinical benefits owing to the tumor intrinsic and extrinsic restriction (primary resistance) [20]. For example, the complex immunosuppressive factors in TME including Tregs and inhibitory checkpoint molecules can restrict T cell infiltration and killing ability, which are critical for tumor eradication [21]. Moreover, tumor intrinsic features including deficient PD-L1 expression, low mutation burden, mismatch repair protein and mutated driver-genes also imparied ICB efficacy [5]. Among all the factors, lack of PD-L1 expression in tumor cells and/or reduced TILs in the stroma are main contributors to ICB resistance, as was widely accepted in the Tumor Immune Microenvironment (TIME) classification [22]. Much effort has been made to overcome these limitations, e.g., development of novel checkpoint targets, engineered T cells, tumor vaccines, targeting other immunocytes, optimizing the predictive biomarkers, and exploring combination strategies [5, 23]. Here we reported a potential novel immunotarget and prognostic biomarker for LUAD patients. We found that enriched ILT4 in tumor cells is correlated with decreased T cell infiltration in the TME and poor patient outcomes. Further analyses revealed that the decreased T cell density is mainly due to impaired CD8^+^T cell frequency. Moreover, high ILT4 expression indicated Treg accumulation in the TME. ILT4 expression in tumor cells combined with CD8^+^T cell/Treg density are stronger indicators for patient outcomes.

ILT4 is a well-established immunosuppressive molecule in myeloid innate cells [10]. Its expression and functionality in the tumor microenvironment have drawn great attention since last decade. We found that enriched ILT4 expression is the common feature for malignancies including NSCLC, breast cancer, hepatocellular carcinoma and colorectal cancer [11, 24–26]. The subsequent investigation from our and other groups demonstrated that tumor cell-derived ILT4 supported tumor growth and metastasis through induction of their malignant behaviors such as proliferation, invasion, migration, EMT and HSC self-renewal [12–14, 27]. More recently, ILT4 was reported to be enriched in TAMs and polarize their M2 phenotype, which resulted in hostile immunological microenvironment [26]. Moreover, ILT4 in activated human CD4^+^ T cells served as Semaphorin-4A receptor to drive Th2 differentiation [28]. These results implicated the immunosuppressive effect of ILT4 in tumor progression. However, the regulation of tumor cell-derived ILT4 on T cell immunity is still unclear. In the current study, we identified that overexpressed ILT4 in tumor cells is correlated with decreased T cell frequency in the TME, as well as advanced diseases and poor patient survival, indicating that tumor cell-derived ILT4 might promote tumor development through restricted T cell infiltration. These findings raised tumor cell-derived ILT4 as a negative prognostic biomarker and immunotherapeutic candicate target for LUAD patients. Blockade of ILT4 using therapeutic monoclonal antibody might act as a useful clinical strategy to inhibit LUAD tumor growth and immune escape. Meanwhile, ILT4 blockade may reverse the immunosuppressive microenvironment which causes ICB resistance, suggesting its role in PD-1/PD-L1 inhibitors-combined therapeutics. More importantly, we observed similar ILT4 expression feature in both EGFR-mutant and -wild-type patients (data not shown). Given that current ICB showed little clinical benefit in EGFR-driven LUAD patients, we supposed that ILT4 blockade might be a potential treatment for this population. All the hypotheses above are under investigation in our group.

We also delineated that ILT4-decreased T cell infiltration is mainly due to diminished CD8^+^ T cell density. CD8^+^T cells play a central role in anti-tumor immune response [29]. They usually destruct tumors directly through release of cytotoxic granules (granzyme and perforin), or indirectly through secretion of cytokines (IFN-γ or TNF) [29]. Almost all studies yield consistent results that CD8^+^ T cells are associated with superior clinical outcomes in NSCLC patients [30, 31]. Nonetheless, there are conflicting results in LUAD, a heterogeneous subtype of NSCLC with totally distinguishing processes and therapies [32, 33]. Here we found CD8^+^T cell accumulation in both cancer nest and stroma of LUAD predicted beneficial clinical outcomes. Based on these results, we proposed that ILT4 might mediate tumor immune escape via inhibition of CD8^+^ T cell traffic and infiltration. In our study, ILT4 did not alter CD4^+^ T cell frequency, possibly due to complex CD4^+^ T subset components and their conflicting effects on anti-tumor immunity [34].

FOXP3 is a lineage-specific transcript factor for Tregs [35]. It not only controls their phenotype, but also maintains their immunosuppressive function, and is the most widely used Treg marker [35]. Treg usually mediates immunosuppressive function through contact-dependent inhibition of T_effertor_, NK or APC activation, or otherwise through secretion of inhibitory factors such as IL-10, TGF-β [36]. Clinically, intra-tumoral Treg accumulation has a detrimental prognostic effect on NSCLC patients [37, 38]. Recently, professor Chen reported TAM-derived ILT4 promoted Treg activation by inducing their inflammatory phenotype [15]. Here we are the first to report that tumor-derived ILT4 might also direct Treg infiltration. We speculated that elevated Treg intensity represents another important mechanism for ILT4-mediated immune escape.

Adaptive T cell immunity prevents tumor development through either direct cell-cell contact or secretion of tumoricidal cytokines [39]. For this reason, intratumorally or stroma infiltrating T cells might have different effects and anti-tumor mechanisms [40]. Recent studies have revealed that spatial architecture of tumor-infiltrating T cells predicted controversial clinical outcomes [41]. Therefore, we assessed ILT4-regulated T cell subset accumulation in both cancer nest and stroma. However, both in cancer nest and stroma, ILT4 expression was similarly connected to tumor-infiltrating CD3^+^T cell/CD8^+^T cell/Treg density. These results suggested that ILT4-regulated T cell infiltration and immunosuppression may not mainly rely on the contact-dependent immunological synapse.

In spite of some inconsistent reports, CD8^+^ T cell and Treg density in the TME are potential prognostic factors for LUAD patients [30, 37, 38, 42, 43]. Our results again confirmed their predictive role for OS. Since ILT4 expression is related with suppressive T subset infiltration, we evaluated the prognostic value of combined ILT4 expression and tumor-infiltrating CD8^+^ T cell/Treg density in LUAD patients. As expected, the combined ILT4 expression and CD8^+^ T/Treg frequency are stronger predictors for patient OS. These results proposed novel and more effective prognostic biomarkers for LUAD patients.

## Conclusion

In conclusion, tumor cell-derived ILT4 is correlated with immunosuppressive T cell subset infiltration and poor clinical outcomes, and might be a potential immunotherapeutic target and prognostic biomarker for LUAD patients. Combined ILT4 expression and CD8^+^ T cell/Treg frequency in TILs are stronger predictors for patient outcomes compared with any of these biomarkers alone.

## Data Availability

All data are included in the article.

## Abbreviations

ILT4: Immunoglobulin-like transcript 4
LUAD: Lung adenocarcinoma
TME: Tumor microenvironment
ICB: Immune checkpoint blockade
LILRB2: Lymphocyte immunoglobulin-like receptor B2
MIR-10: Monocyte/macrophage immunoglobulin-like receptor 10
DC: Dendritic cell
NSCLC: Non-small cell lung cancer
EMT: Epithelial-mesenchymal transition
PIR-B: Paired Ig-like receptor
MDSC: Myeloid-derived suppressor cells
TAM: Tumor-associated macrophage
HSC: Hemopoietic stem cell
Treg: Regulatory T cell
UICC: Union for International Cancer Control
GEO: Gene Expression Omnibus database
TCGA: The Cancer Genome Atlas
PFS: Progression free survival
OS: Overall survival
TIL: Tumor infiltrating lymphocyte
HR: Hazard rate
TIME: Tumor immune microenvironment
